# Is There a “Biological Desert” With the Discovery of New Plant Viruses? A Retrospective Analysis for New Fruit Tree Viruses

**DOI:** 10.3389/fmicb.2020.592816

**Published:** 2020-11-19

**Authors:** Wanying Hou, Shifang Li, Sebastien Massart

**Affiliations:** ^1^Key Laboratory of Tobacco Pest Monitoring Controlling and Integrated Management, Institute of Tobacco Research, Chinese Academy of Agricultural Sciences, Qingdao, China; ^2^Plant Pathology Laboratory, TERRA, Gembloux Agro-Bio Tech, University of Liège, Gembloux, Belgium; ^3^Environment and Plant Protection Institute, Chinese Academy of Tropical Agricultural Sciences, Haikou, China; ^4^State Key Laboratory for Biology of Plant Diseases and Insect Pests, Institute of Plant Protection, Chinese Academy of Agricultural Sciences, Beijing, China

**Keywords:** virus diseases, biological characterization, fruit trees, new virus, potential risks

## Abstract

High throughput sequencing technologies accelerated the pace of discovery and identification of new viral species. Nevertheless, biological characterization of a new virus is a complex and long process, which can hardly follow the current pace of virus discovery. This review has analyzed 78 publications of new viruses and viroids discovered from 32 fruit tree species since 2011. The scientific biological information useful for a pest risk assessment and published together with the discovery of a new fruit tree virus or viroid has been analyzed. In addition, the 933 publications citing at least one of these original publications were reviewed, focusing on the biology-related information provided. In the original publications, the scientific information provided was the development of a detection test (94%), whole-genome sequence including UTRs (92%), local and large-scale epidemiological surveys (68%), infectivity and indicators experiments (50%), association with symptoms (25%), host range infection (23%), and natural vector identification (8%). The publication of a new virus is cited 2.8 times per year on average. Only 18% of the citations reported information on the biology or geographical repartition of the new viruses. These citing publications improved the new virus characterization by identifying the virus in a new country or continent, determining a new host, developing a new diagnostic test, studying genome or gene diversity, or by studying the transmission. Based on the gathered scientific information on the virus biology, the fulfillment of a recently proposed framework has been evaluated. A baseline prioritization approach for publishing a new plant virus is proposed for proper assessment of the potential risks caused by a newly identified fruit tree virus.

## Introduction

The fruit trees are high-value crops grown worldwide. Stone, pome, citrus, rubus, ribes, blueberry mulberry, kiwifruit, and persimmon fruit trees represent the major cultivated species. Nevertheless, numerous plant viruses can infect them, sometimes at a very high prevalence. The virus infection can originate from vegetative propagation and grafting of infected cultivars, and might be exacerbated during the perennial life cycle by horizontal transmission accelerating the mixing and infection of viruses of individual plants (Czotter et al., 2018). Some of these pathogens cause severe crop losses and often reduce the productive life of the orchards. For example, the plum pox virus (PPV, genus *Potyvirus*) causes “sharka” disease, the most devastating viral disease of stone fruit trees worldwide, which causes severe damages, estimating a total cost at 10 billion Euros worldwide in 30 years (García et al., 2014; Rimbaud et al., 2015). Citrus tristeza virus (CTV; genus *Closterovirus*) is probably the most economically important virus infecting citrus, causing a decline of sour orange rootstock, yellow seedling of lemon and grapefruit, and stem pitting in grapefruit and sweet orange (Moreno et al., 2008; Umer et al., 2019). The disease has led to the death of millions of citrus trees all over the world and has rendered millions of others useless for production (Lee, 2015).

The identification, detection, and characterization of the causal agent(s) of viral disease symptoms could be challenging, due to the low titer of several viruses, their heterogeneous distribution between tissues or over time within the tree, the frequent mixed infections, the absence of universal primers for the detection of all viruses, the occurrence of symptomless infection, and the impact of the cultivar on the symptom development (Czotter et al., 2018; Maliogka et al., 2018). Another challenge for a complete viral indexing of a diseased tree was the intrinsic genome variability of plant viruses, which complicates the design of inclusive primers able to detect any isolate of a species or a specific genus or family through classical molecular test (Massart et al., 2014). Therefore, it was required to combine several tests like electron microscopy, serological or molecular techniques, and biological assays on indicator plants to achieve a complete indexing and the identification of viral species infecting the diseased trees with symptoms of unknown etiology.

The advent of high throughput sequencing (HTS) technologies during the last decade has dramatically changed research on viral and virus-like agents. HTS technologies are a potential universal screening method for plant virus detection, allowing for the theoretical detection and identification of any known or unknown agents. Until recently, there were many fruit tree diseases with unknown etiology, although a viral origin was suspected. Nevertheless, the development of HTS technologies has drastically changed the situation (Zheng et al., 2017). In fact, they have accelerated the pace of discovery and identification of new viral species and the characterization of their genome (Massart et al., 2014). As an example, until 2011, the genomes of 50 different viruses from pome and stone fruit species were sequenced (Martelli et al., 2011), representing an average of 1.2 viruses per year since the development of sequencing technologies. From 2012 to 2016, three and seven new viruses were found in pome and stone fruits (Rubio et al., 2017), respectively, (average: 2 per year). From 2017, the number of newly identified viruses reached 28, representing an average of 9.3 novel species per year. Up to now, HTS technologies allowed the identification and genome characterization of nearly 40 novel viruses from pome and stone fruits in a few years.

The pace of virus discovery since the advent of HTS technologies also raises an important question: How do virologists address the biological characterization of new viruses with the genome sequence information as a starting point? Indeed, these new viruses identified by HTS technologies are often lacking information on their biology and the risk they can pose on fruit production. This is an important concern for carrying out a proper risk assessment. For example, a panel of experts highlighted in a recent report that for several viruses, especially those recently discovered, the pest categorization based on pest risk analysis is associated with high uncertainties due to the absence of data on biology and distribution (Bragard et al., 2019a). Biological characterization of a new virus is a complex and lengthy process requiring comprehensive knowledge on epidemic potential, possible alternative hosts in ecosystems, symptomatology on various cultivars and host species, vectors and modes of transmission, geographical distribution, and interactions with other viruses (Massart et al., 2017). Definitively linking a novel pathogen candidate with observed disease symptoms, according to Koch’s postulates, is not easy or sometimes turns out to be impossible for some viruses. Koch’s postulates are based on the one pathogen–one disease paradigm of infection biology and are inadequate in cases of diseases with polymicrobial causes. Therefore, the suitability of alternate strategies based on epidemiological observations and appropriate statistics for determining causal relationships of disease have been proposed when other experimental demonstrations of causation cannot be readily achieved (Fox, 2020).

A decade after the first discovery of a phytovirus by HTS technologies (Kreuze et al., 2009), it is now scientifically relevant and timely to address important questions related to the identification of new fruit tree viruses: When a new virus is discovered by HTS technologies, what information is published? To which extent do the scientists explore the biology when publishing a newly discovered virus: genome variability, prevalence, transmission, and host range?

After the original publication reporting the discovery of a new fruit tree virus, the high pace of virus discoveries also raises another question: Once a new virus is discovered and published, does it trigger additional experiments or surveys by the virologists to complete its biological characterization? Indeed, resources are limited for plant virologists, and the current abundance of newly identified viruses might limit their downstream biological characterization. This publication also evaluated how a recently proposed framework for the evaluation of biosecurity, commercial, regulatory, and scientific impacts of plant viruses and viroids identified by HTS technologies is fulfilled (Massart et al., 2017).

The objectives of this publication were, therefore, to critically review and perform and in depth analysis of (1) the scientific information on the virus biology that was published together with the genome sequence when a fruit tree virus was discovered, and (2) the content of the scientific publications citing the original publication reporting the first publication of a fruit tree virus. We focus our analysis on the newly discovered viruses since 2011 and from the major fruit tree species worldwide. The gathered information was categorized, and general conclusions are provided. Overall, this analysis contributes to the establishment of a baseline and prioritization of complementary experiments to be done once a new fruit tree virus is discovered in the near future.

## Screening of the Scientific Literature and Categorization of Experiments

### Analysis of the Publications Reporting a New Fruit Tree Virus

Thirty-two fruit tree species which listed in identified host species in Supplementary Table 1 were included in our analysis. The NCBI nucleotide database was surveyed with the keywords of common name of the fruit tree species to identify virus or viroid species submitted for the first time since January 1, 2011. A first list of virus names was elaborated, and the corresponding publications retrieved. In addition, a literature survey was also carried out using Scopus with the keywords of the virus names retrieved from NCBI to complete the publication list.

All the publications reporting the discovery of a new virus or viroid species from January 1, 2011 to April 1, 2020 were screened. The information provided for the characterization of the new viral species has been classified into 13 categories:

– Complete genome: whole viral genome has been sequenced, including the UTR region at the 3′- and 5′-terminus.
– Primer design: virus-specific oligonucleotide primers and (RT)-PCR protocol were designed for RT-PCR detection and are described in the publication or the Supplementary Material.
– Genome diversity: several complete or near-complete genome sequences from different isolates were published and compared.
– Gene diversity: several partial or complete gene sequences (like the polymerase or the coat protein) were sequenced and aligned.
– Local survey: after the discovery, samples were collected in the same location (either a commercial orchard, research station, or germplasm collection) to evaluate the prevalence of the virus.
– Large scale survey: after the discovery, samples were collected from different locations, producing regions in one country or different countries.
– Association with symptoms: whatever the scale of the survey, the sampling was carried out on symptomatic and asymptomatic trees to evaluate the association between the virus presence and symptoms.
– Co-infection with other viruses: samples infected by a new virus are also infected by other known viruses which have been checked by RT-PCR.
– Infectivity bioassays: inoculation of plants using an infectious clone or graft inoculation to a host from the same cultivar or different cultivars of fruit tree species.
– Indicators: transmission to several herbaceous indicators were attempted, even if not successful.
– Symptomatology: symptoms have been observed on the grafted plants in a greenhouse experiment or grafted plants, including host plants and indicators, which have been mechanically inoculated with sap from symptomatic samples or transfers by a natural vector.
– Transmission: at least one natural vector (mite, aphid) has been identified. It might have been used for transmission assays.
– Host range: the virus has been detected on at least another plant species during a survey or successfully inoculated to another plant species (not an indicator).

### Analysis of the Peer Review Articles Citing the Publication of a New Fruit Tree Virus

The peer-review publications citing one of the publications describing a newly identified fruit tree virus were also reviewed and analyzed in depth. They were retrieved based on the Scopus citations. First, the citation of each publication reporting the discovery of a new fruit tree virus was analyzed as follows: the total number of citations and number of citations per year from the initial publication. The information provided in the citing publication was classified into 18 categories in Supplementary Table 2. Nine categories brought additional information on the biological characterization (transmission, survey, and genome diversity) or new host and new country reported of the new virus, while other nine categories are not related to the biological or geographical characterization of the new virus.

## Publications of New Fruit Tree Viruses From 2011

A total of 78 scientific peer-review publications describing the discovery of a new virus or viroid species in the studied fruit tree species were identified between January 1, 2011 to April 1, 2020. These publications reported 81 new virus and three new viroid species among which 13 viruses and 2 viroids for pome fruit species, 22 viruses for stone fruit species, 14 viruses for *Citrus* sp., 8 viruses for *Ribes* sp., 6 viruses for *Rubus* sp., 17 viruses and one viroid for other minor fruit trees. The full name and abbreviations of all these new virus species is listed in Supplementary Tables 1–3.

For the most identified fruit tree species, 15 viruses from apple trees and thirteen viruses from sweet orange, at the opposite only 2 from pear, nectarine and lemon trees while a single virus from pear, Japanese pear, David’s peach, Japanese apricot, plum, raspberry and, American blackcurrant. 13 viruses (TFDaV, CPrV, PrVT, CTLaV, ChALV, PrVF, PrGVA, CVF, CVTR, CCGaV, CiVA, BcLRaV-1, and BCCV-1) identified from more than one host species in the first publications.

## Analysis of the Scientific Information Added when a New Viral Species Is Discovered

The scientific information provided in the original publication describing a new virus is highly variable. The minimal information corresponded to a recent publication of HTS sequences gathered into contigs without any biology confirmation of the results (Wright et al., 2020). On the other hand, a comprehensive biological characterization of the newly identified viruses was provided for temperate fruit decay associated virus (TFDaV). In a publication regarding TFDaV, 45 complete viral genomes of TFDaV were sequenced and analyzed from different host species: apple, pear, and grapevine. Samples displaying virus-like symptoms collected at different regions of the country were evaluated by PCR and rolling-circle amplification (RCA). The ability of TFDaV to infect apple and pear tree seedlings and to cause growth reduction was confirmed by infectivity tests using the cloned viral genome (Basso et al., 2015). Another example is HTS applied to the citrus yellow mottle-associated virus (CiYMaV), which had been discovered from field samples that mainly showed virus-like symptoms. After characterizing the genome of CiYMaV, several aspects of its biology had been evaluated, including host range, symptomatology, association with symptoms, and epidemiology. Bioassays were performed by graft- and mechanical inoculation on eight citrus species and seven herbaceous species, symptoms of oak-leaf pattern and vein yellowing was observed, with CiYMaV detected in all symptomatic plants. The full CP gene of CiYMaV was amplified using a specific primer pair to study sequence diversity. A detection method was designed specifically for CiYMaV and revealed high prevalence (62%) in 120 citrus trees from the Punjab Province in Pakistan, where the novel virus was found mainly in mixed infections with citrus yellow vein clearing virus (CYVCV; 45%) or CTV (9.2%). However, a preliminary survey on samples from 200 citrus trees from the Yunnan province in China failed to detect CiYMaV in this region (Wu et al., 2020).

**FIGURE 1:**
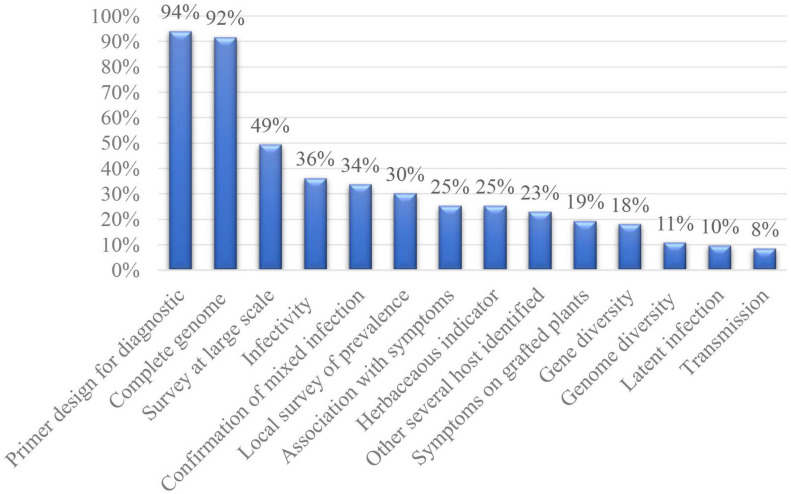
Scientific information added when a new fruit tree viral species is discovered.

Our analysis showed that almost all of the discovered genomes (76 viruses, 92%; Figure 1) were amplified, cloned, and Sanger sequenced, including the UTR regions. The complete genome sequence retrieved was not confirmed by Sanger sequencing for only seven viral species, including PcVT and CVF, which had almost the whole genome sequenced but lacked UTR regions (Jo et al., 2018; Koloniuk et al., 2018a). ARWaV-1, ARWaV-2, IrCRSaV, CJLV, and CVLV had only sequenced partial fragments published (Sadeghi et al., 2016; Matsumura et al., 2017; Wright et al., 2018). Until recently, there was no reporting of unconfirmed HTS sequences without any characterization (Wright et al., 2020).

Detection primers based on the assembled sequences were designed for 78 viruses (94%). The primers were only used to confirm the virus presence in the original sample or experimentally inoculated plants for 32 new viruses. For example, the detection primers designed for CJLV and CVLV had only been used to confirm the presence of the viruses identified in the RNA-seq and sRNA libraries (Matsumura et al., 2017). The PCR-based protocols were applied in the frame of local or large-scale surveys for 57 new viruses. Several primers or protocols were designed and tested for 14 new viruses. Nineteen publications gave little importance to these primers as their sequences were described in the Supplementary Material.

More than a single genome was sequenced for nine viruses (11%). In this case, the genome diversity was always analyzed. A maximum of 45 complete viral genomes of TFDaV were sequenced, including 17 from apple, 26 from pear and two from grapevine (Basso et al., 2015). On the other hand, a complete genome of two isolates of peach-associated luteovirus (PaLV) were obtained and compared (Wu et al., 2017). In total, three publications reported genomes of isolates from very distant geographical locations (Martin et al., 2011; Marais et al., 2015; Koloniuk et al., 2018b). For three viruses, the genome of isolates was obtained and compared in different host species. One is TFDaV, and the other two are PrVT and PrGVA (Al Rwahnih et al., 2018).

The diversity has been analyzed at genes or partial gene levels for 15 (18%) new viruses. The comparison was based on partial genome sequences obtained by classical Sanger sequencing. The sequenced fragment ranged from a partial gene to several genes. For apricot vein clearing-associated virus (AVCaV), PCR amplicons of 330 bp from the replicase gene were obtained from two samples (Elbeaino et al., 2014). In contrast, the nucleotide sequence of the entire CP from 58 different CiYMaV isolates was determined and aligned (Wu et al., 2020). The population diversity of blackberry vein banding-associated virus (BVBaV) was studied on 25 isolates for three genes (Thekke-Veetil et al., 2013).

A local survey of prevalence was carried out for 25 (30%) new species. The number of tested samples were also variable, ranging from four to more than 200. For example, four sweet cherry trees maintained at the Yamagata horticultural experiment station were evaluated to the study ChVB (Yaegashi et al., 2020). On the other hand, the local survey incidence of PrGVA included 215 samples collected from the National Clonal Germplasm Repository, selected trees represented a diverse array of *Prunus* sp. (Al Rwahnih et al., 2018). Tested samples usually ranged from 10 to 100 in other species. Less than 10 samples were tested for five viruses: NSPaV, ChVB, PeVB, PVd2, and BCI. Two (PrGVA, BCCV-1) had been tested in different host species.

The discovery was completed by a large-scale survey, including samples collected from different locations for 41 (49%) new species. The extent of the survey was highly variable, ranging from tens to hundreds of samples. For example, in a publication with cherry virus F (CVF), a small survey on nine cherry trees form four locations in different countries was carried out (Koloniuk et al., 2018a). Moreover, 524 samples collected from several areas in the United States were surveyed for the presence of blueberry mosaic associated virus (BLMaV) in both wild and cultivated trees (Hassan et al., 2017). In the large scale survey on the prevalence of Mume virus A (MuVA), a total of 285 samples from 11 *Prunus* sp. trees from China, Japan, Czech Republic, Azerbaijan, Kazakhstan, Italy, and France were screened (Marais et al., 2018). 80 peach samples collected from two germplasm nurseries located in different provinces in China were tested for the incidence of PLPaV (He et al., 2017). In total, more than one hundred samples were surveyed for 18 species, and less than 30 samples tested in five species. Eleven species (ARWV-1, ARWV-2, TFDaV, CPrV, PrVT, PrVF, MuVA, CVTR, CCGaV, CiVA, and BCCV-1) were tested for different host species, while 12 (AGCaV, ApNMV, CPrV, PrVT, MuVA, CVF, CRV-5, CiYMaV, RLBV, BlMaV, BFDaV, and MBV1) were tested for samples collected from different countries.

The sampling was carried out on symptomatic and asymptomatic trees to evaluate the association between the virus presence and symptoms in 56 (67%) species, including 15 at a local scale, 10 both at a local and large scale, and 31 at a large scale. Depending on the survey results, a high association with symptoms was found for 21 (25%) virus species: AGCaV, ApNMV, ARWV-1, ARWV-2, ARWaV-1, ARWaV-2, CCGaV, CYVCV, CiLV-C2, IrCRSaV, CiLV-N, CiYMaV, RLBV, BVBaV, BLMaV, BNRBV, BlMaV, BFDaV, MMDaV, AcCRaV, and AcEV-2. A possible latent infection had been concluded for eight (10%) species: AGV, AaLV, PrGVA, BBLV, BLSV, BVA, PeCV, and ASbLV. An association between symptoms and virus infection were confused in 27 (32%) species.

The association with symptoms in 25% species based on much higher incidence in symptomatic plants than in asymptomatic plants, regardless of the scale of the survey. For example, the incidence of ApNMV was 83% in symptomatic samples and 37% in asymptomatic samples from 359 samples in a survey of mosaic-diseased apple trees from major apple-producing provinces in China (Noda et al., 2017). CYVCV was considered to be highly associated with symptoms, according to the results from seven samples: four symptomatic plants and three asymptomatic plants from a local survey (Loconsole et al., 2012a). AcEV-2 was identified from a kiwifruit tree showing leaf mottle and chlorosis symptoms. Meanwhile, most AcEV-2-infected kiwifruit trees showed viral disease-like symptoms (Wang et al., 2020).

Latent infection was found in 10% species, and this was based on most of the positive samples that were symptomless. For example, PeCV had a high incidence in symptomless plants, while the original symptoms (vein necrosis) could not be linked to its presence (Morelli et al., 2015). BLSV was also considered to be latent as it did not cause any obvious symptoms in the highbush blueberry in a single infection (Isogai et al., 2012).

The association between symptoms and virus infection remained unclear in 32% species after a large-scale survey due to several reasons. The low incidence (<1%) or absence of virus (such as CPrV, MuVA, PeV1, AVCaV, CRV-5, PLPaV, and PVd2) in the survey impeded any association based on sufficient number of samples. Another limitation of the association is the presence of other viruses in the infected trees. This phenomenon was reported for 12 species (TFDaV, MdoVA, ChALV, PaLV, PcVT, ChVB, BVF, BCIV, BCaRV, BCCV-1, PrVT, and PeVB) and underlined the need to complete the survey on the newly identified virus with other known viruses. Symptom variability can also hamper the establishment of an association in eight species (ALV-1, NSPaV, NeVM, CVF, PrVF, CiVA, CVTR, and MBV1).

The presence of co-infecting viruses was reported and confirmed by RT-PCR for 28 (34%) viral species. Among them, a prevalence survey of four (AGCaV, ALV-1, CYVCV, and AcEV-2) was carried out surveying for other co-infecting viruses. Another 24 species mentioned co-infection in the original sequenced plant but did not survey for another co-infecting virus or report the names of the co-infecting virus species.

Infectivity bioassays in the correspondent hosts were carried out for 30 (36%) viral species. All of the bioassays resulted in positive RT-PCR detection, and 12 reported observed symptoms. Infectious clones were constructed for TFDaV, consisting of circular single-stranded DNA of family *Geminiviridae* and AHVd belonging to *Pelamoviroid*. Growth reduction was observed in apple and pear plants following biolistic inoculation with the cloned TFDaV (Basso et al., 2015). But, no symptoms were observed in AHVd clone inoculated plants (Serra et al., 2018). CiLV-C2, CiLV-N, and RLBV were successfully transmitted by mites, while 25 others were only successfully inoculated by grafting. For BLSV, six cultivars were successfully infected through graft transmissions; meanwhile, mechanical inoculation with purified BLSV particle into forty seedlings of highbush blueberry failed (Isogai et al., 2012). Three publications (CCDaV, CiYMaV, and PLPaV) confirmed the presence of co-infecting viruses on grafted plants (Loconsole et al., 2012b; He et al., 2017; Wu et al., 2020). Eight publications did not mention weather another virus along with the studied one (NSPaV, CCDaV, CYVCV, CiLV-C2, CVEV, IrCRSaV, CiLV-N, and RLBV).

Transmission to herbaceous indicators have been attempted for 21 viruses (25%). More than one herbaceous plant species was inoculated for 13 species. Ten transmissions allowed the observation of symptoms. However, nine failed to inoculate (TFDaV, PrVF, MuVA, PrGVA, ChVB, BLMaV, BVA, BFDaV, and PeCV). Two species (AGV and BVF) had successful infections, although none of the infected plants displayed visible symptoms. Among the 10 species with observed symptoms, CiLV-N and CiCSV were transmitted by mites while eight (PLPaV, CYVCV, IrCRSaV, CiYMaV, RLBV, BCLCaV, BLSV, and AcCRaV) were mechanically transmitted. Among the nine failed experiments, only TFDaV was biolistically-inoculated with the *Spe*I-linearized and recircularized genome onto *Nicotiana benthamiana* (Basso et al., 2015). The other eight failed experiments were mechanical inoculation.

Symptoms have been observed for 16 (19%) viral species. These including symptoms observed on both grafted host plants and herbaceous indicators in six (PLPaV, CYVCV, IrCRSaV, CiLV-N, CiYMaV, and RLBV), only observed on experimental host plants in six (TFDaV, NSPaV, CCDaV, CiLV-C2, CVEV, and CCGaV), and only on herbaceous indicators plants in four (AcCRaV, BLSV, BCLCaV, and CiCSV).

More than one host species within the trees studied was identified for 19 (23%) viruses. 13 (TFDaV, CPrV, PrVT, CTLaV, ChALV, PrVF, PrGVA, CVF, CVTR, CCGaV, CiVA, BcLRaV-1, and BCCV-1) were naturally infected in different host species identified, and six (MuVA, CCDaV, CYVCV, CVEV, IrCRSaV, and CiYMaV) were successfully inoculated to another plant species in infectivity bioassays. Three species (CCGaV, CiVA, and PrGVA) that were identified in different host species naturally were also infectable while inoculated on another plant species (Navarro et al., 2018). Only TFDaV was found to naturally infect different host species which belong to different genera, while another 18 infected different host species belonging to the same genus.

The possible natural transmission mode and vectors were investigated for 7 (8%) viral species. Among them, potential natural vectors have been identified for five species (CiLV-C2, CiLV-N, CiCSV, RLBV, and BVA). Four of them successfully transmitted from one host plant to another through a natural vector (mite or aphid) while one (BVA) failed with cotton aphids (Isogai et al., 2013). Only BBLV tested seed transmission and BCLCaV mentioned pollen transmission (Martin et al., 2011; James and Phelan, 2017).

Seventeen viruses (20%), including PpPV2, ApRVA, PeVD, PcVT, PCLSV, PYSaV, CJLV, CVLV, CTNGmV-1, CTNGmV-2, BVE, CuLV, CuVA, RAVA, BGMaV, PeLV, and AcV-1 lacked information regarding the biological characterization of epidemiological survey, infectivity or indicator assays, symptomatology, transmission, and host range. However, they did have genetic annotations or information regarding detection confirmation when first published.

## Biology Progress After the Discovery of New Fruit Tree Virus

The 78 original publications of a new fruit tree virus have been cited 933 times (Supplementary Table 2), representing an average of 12 citations per publication. If these numbers are reported per year from the initial publication, the average citation is 2.8 per year. The most frequently cited publication was on CCDaV, which had been cited by 70 other peer-review publications since 2012, with an average of 8.8 citations per year (Loconsole et al., 2012b).

**FIGURE 2:**
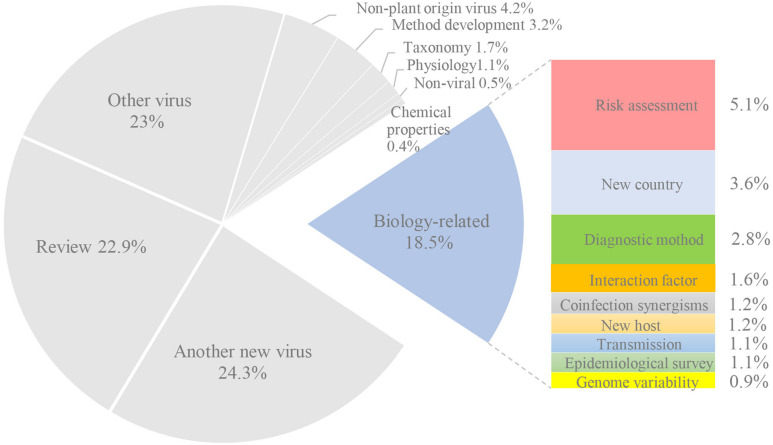
Publications citing a new fruit tree viral species.

The percentages (Figure 2) for each category calculated from Supplementary Table 2. Only 18% of citation covered information on the biology or geographical repartition of the newly discovered viruses. This is an average of two citations per new virus (and a yearly number of 0.5). Among them, 3.6% and 1.2% reported the presence in a new country or host, respectively. The development of a diagnostic method was mentioned in 2.8% of the citations, while 0.9% studied genome variability by sequencing new isolates. Only 1.1% of the citations corresponded to epidemiological surveys, and another 1.1% focused on transmission. Finally, 1.6% focused on the interaction factor between virus and host, and 5.1% were related to risk assessment. The most cited for biology is a publication on CYVCV which was first identified as the putative viral causal agent of yellow vein clearing disease (YVCD) in lemon trees in Pakistan (Loconsole et al., 2012a). In total, 22 publications (43%) were cited by publications on the biology of CYVCV. Five publications reported CYVCV in new countries and new host. Six publications developed diagnostic methods for CYVCV and four identified transmission vectors. Four publications focused on epidemiology and variability, and monitoring of CYVCV in China indicated a low level of sequence heterogeneity among CYVCV isolates of different geographic origins and hosts (Zhou et al., 2017). The interaction factor of and host response to CYVCV was also further investigated in three publications.

The other nine categories, which were not related to the biological or geographical characterization of a new virus, corresponded to 82% of all citations. Nearly half of the citations corresponded to reviews (22.9%) or the discovery of another virus (24.3%). In addition, 23% were related to the study of a known virus. The other categories represented less than 5%. Most of the citing publications were, therefore, not related to virus biology and repartition. Moreover, they did not provide useful information to better evaluate the phytosanitary risks posed by the new viruses.

The detection of the new virus in another country on the same continent was reported for six species: ApNMV, CYVCV, CCDaV, CRMaV, RLBV, and PeCV. ApNMV was identified in Japan and reported from China and Korea (Cho et al., 2017; Xing et al., 2018). CYVCV was identified from Turkey, and reported in China, Pakistan, and India (Chen et al., 2014; Cao et al., 2016; Yu et al., 2017; Meena et al., 2019). CCDaV, identified from Turkey, was reported in China (Guo et al., 2015; Karanfil and Korkmaz, 2019). CRMaV was identified from Canada but also reported in South Carolina (Poudel and Scott, 2017). RLBV, identified from Scotland, was reported in Montenegro, Bulgaria, Finland, and Serbia (Mavriè Pleško et al., 2014; Zindović et al., 2015; Dong et al., 2016; Jevremović et al., 2019). PeCV, identified from Italy, was also reported in North Macedonia, Spain, and Turkey (Morelli et al., 2015; Morelli and Arli-Sokmen, 2016; Ruiz-García et al., 2017; Jevremović and Paunović, 2019).

A new virus was detected in another continent for 14 species: AGCaV, AHVd, ARWV-1, ALV-1, NSPaV, CVF, ChALV, PrVF, PaLV, CVEV, BlMaV, PeVA, MBV1, and AcV-1. AGCaV, identified from Australia and Canada, was reported in Korea (Cho et al., 2016). AHVd had been reported in the United States, Japan, Italy, Spain, and New Zealand since it had been identified as a new viroid species (Szostek et al., 2018; Chiumenti et al., 2019; Sanderson and James, 2019). ALV-1 and ARWV-1, identified from the United States, were confirmed to be co-infected with more than two other viruses in apple rootstocks in Korea (Lim et al., 2019). NSPaV, discovered from nectarine cultivars imported from France to California, was reported in Italy, China, South Korea, and Japan (Candresse et al., 2017; Igori et al., 2017a; Lu et al., 2017; Sorrentino et al., 2018). CVF, identified from The Czech Republic and Greece, was reported in Canada (James et al., 2019). ChALV, identified from The Czech Republic, was reported in South Korea (Igori et al., 2017b). PrVF had been reported in Canada, Belgium, and The Czech Republic since it was first identified (Šafáøová et al., 2017; James et al., 2018a; Tahzima et al., 2019). PaLV, identified from the USDA Appalachian Fruit Research Station (ARS) in West Virginia, was reported in Italy and China (Sorrentino et al., 2018; Zhou et al., 2018). CVEV, identified from Spain, was reported in Japan and China (Huang et al., 2015; Nakazono-Nagaoka et al., 2017). BlMaV was confirmed from three states in the United States and British Columbia, and Canada, and reported in Japan and Serbia (Thekke-Veetil et al., 2014; Jevremović et al., 2015; Isogai et al., 2016). PeVA was identified from Japan and also reported in Italy (Ito et al., 2013). MBV-1 was identified in mulberry from Lebanon, Turkey, and Italy, but also reported in Iran (Alishiri et al., 2016). AcV-1, identified from Italy, was reported in China (Blouin et al., 2018; Wen et al., 2019).

A new host was identified for nine species: AGCaV, ApNMV, AVCaV, NSPaV, ChALV, PrVF, CYVCV, CCGaV, CiVA, CiCSV, and CiLV-C2. A new strain of AGCaV, which was identified from apple trees, was first reported in quince (*Cydonia oblonga*; Morelli et al., 2017). ApNMV, which was isolated from apple, was reported in crabapple (*Malus.*spp; Hu et al., 2019). AVCaV only infected one out of 39 varieties of apricot while identified, but was reported in four additional species (*P. salicina, P. mume, P. domestica*, and *P. persica*; Abou Kubaa et al., 2014; Kinoti et al., 2017). NSPaV was identified in nectarine trees and reported to infect peach and Japanese apricot trees (Candresse et al., 2017; Igori et al., 2017a; Lu et al., 2017; Sorrentino et al., 2018). A highly divergent South Korean (SK) isolate of ChALV, which was identified from sweet and sour cherry trees, was also reported in peach trees (Igori et al., 2017b). PrVF, identified from three *Prunus* species (*P. avium, P. domestica*, and *P. cerasifera*), was reported in a new natural host, sour cherry (*P. cerasus*; Koloniuk et al., 2018a). CYVCV, identified in lemon trees, proved to naturally infect different citrus (Kinnow mandarin, sweet oranges) and weed species (Önelge et al., 2016; Meena et al., 2019). CCGaV was identified from citrus trees and reported in apple trees (Wright et al., 2018). CiVA was identified in citrus and then reported in pear (Svanella-Dumas et al., 2019). Besides affecting sweet orange CiCSV also infects *Talipariti tiliaceum* and *Agave desmettiana* (Chabi-Jesus et al., 2018; Chabi-Jesus et al., 2019). CiLV-C2 has been reported naturally infecting *Hibiscus rosa-sinensis*, *Dieffenbachia* sp., and *Swinglea glutinosa* (Roy et al., 2015, 2018).

A high association with symptoms, based on the survey results on the incidence in symptomatic and asymptomatic plants in the first publication, was confirmed for six species (ApNMV, AGCaV, NSPaV, CYVCV, RLBV, BlMaV, and CCGaV) when reported in a new country or host. ApNMV, which was first correlated with the apple mosaic symptoms in Japan, was also reported at a high incidence in apple trees with mosaic symptoms in China and Korea (Cho et al., 2017; Xing et al., 2018). AGCaV, which was associated with apple green crinkle disease (AGCD) in Australia was reported in apples showing severe symptoms of AGCD in Canada, and reported to co-infect with other malus viruses in apples, showing small leaves or growth retardation in Korea, and in a new host quince with severe disease (Cho et al., 2016; Morelli et al., 2017). CYVCV was first described as the putative viral causal agent of YVCD in lemon trees from Turkey, and was also associated with YVCD in other countries and other citrus species (Chen et al., 2014; Cao et al., 2016; Yu et al., 2017; Meena et al., 2019). RLBV was associated with symptoms of leaf blotch disorder in raspberry plants and reported in samples that showed virus-like symptoms (including chlorotic mottling and yellow blotches) in four counties. BlMaV, which was confirmed in mosaic samples collected from three states in the United States and British Columbia, Canada, was later reportedly associated with blueberry mosaic disease (BMD) in Japan and Serbia. CCGaV, identified to have an association with citrus concave gum disease (CG) affecting citrus trees, was found at a high incidence in apple-decline affected trees (Wright et al., 2018).

Six publications focused on transmission related to four species: CYVCV, CiLV-C2, CiLV-N, and BNRBV. Four citing publications identified the transmission vector of CYVCV (Zhou et al., 2015; Zhang et al., 2018; Zhang Y.H. et al., 2019; Zhang Y. et al., 2019). One publication related to CiLV-C2 studied transmission (León et al., 2017). One publication was conducted to determine how BNRBV spreads in the field (Robinson et al., 2016).

Eight publications focused on genome diversity related to six species: AHVd, CYVCV, BCLCaV, BlMaV, MBV1, and AcV-1. Two publications analyzed the genetic diversity of genome sequences from variants of AHVd (Chiumenti et al., 2019; Sanderson and James, 2019). Genetic stability among CYVCV isolates from different geographic origins were analyzed (Zhen et al., 2015; Chen et al., 2017). Analysis of HTS derived paired-end reads revealed the existence of bridge reads encompassing the 3′- and 5′-terminus of RNA-2 or RNA-3 of BCLCaV (James et al., 2018b). The genome diversity of BlMaV was examined using 61 isolates collected from North America and Slovenia (Thekke-Veetil et al., 2015). One publication characterized MBV1-derived small RNAs (Chiumenti et al., 2016). One publication compared CP sequences of different AcV-1 isolates with an isolate that was reported in New Zealand, and showed sequences identities of CP nucleotides and amino acids among these isolates, which were 84.8–97.1% and 89.7–99.6%, respectively, (Peng et al., 2020).

Sixteen publications focused on the diagnostic method for 14 species: AGCaV, NSPaV, PaLV, PeVD, CCDaV, CYVCV, CiLV-C2, CVEV, BE, BVBaV, BBLV, BLSV, BNRBV, and BlMaV. Real-time RT-PCR assays were developed for two viruses (ASGV and AGCaV) infecting pome fruit (Beaver-Kanuya et al., 2019). Multiplex RT-PCR was developed to simultaneously detect three new viruses (NSPaV, PaLV, and PeVD) that infect peach (Xu et al., 2019). A loop-mediated isothermal amplification assay was established for CCDaV (Liu K. et al., 2017). Six publications developed diagnostic methods for CYVCV (Chen et al., 2016; Liu Z. et al., 2017; Liu et al., 2019; Zhao et al., 2017; Bin et al., 2018; Meena et al., 2020), and four developed diagnostic methods for CiLV-C2 (Choudhary et al., 2013, 2014, 2015, 2017). A quantitative RT-PCR approach for the quantification of CVEV was also developed (Wang et al., 2016). One publication describes methods for the extraction of nucleic acids for molecular testing from a range of different berry fruit crops, and lists oligonucleotide primers that were developed to amplify a large number of berry fruit viruses related to BVE, BBLV, BNRBV, and BlMaV (Macfarlane et al., 2015). One publication focused on reliable detection assays for BlMaV (Thekke-Veetil and Tzanetakis, 2017).

Six publications focused on epidemiological surveys related to nine species: ApNMV, CCDaV, CYVCV, BVE, BLMaV, BBLV, BlMaV, BFDaV, and AcCRaV. A large scope epidemiological survey in China demonstrated that ApNMV was highly associated with mosaic disease in apple trees (Xing et al., 2018). One publication investigated the potential spread of CCDaV in commercial orchards, and showed that Turkish and Chinese samples clustered into different groups (Karanfil and Korkmaz, 2019). Monitoring the presence of CYVCV in China indicated that there is a low level of sequence heterogeneity among CYVCV isolates from different geographic origins and hosts (Zhou et al., 2017). The incidence of BVE and BLMaV in two large-scale blackberry plantings in South Carolina demonstrated the transmission of mites (Poudel et al., 2018). A survey for blueberry viruses was carried out in the United States and included BBLV, BlMaV, and BFDaV (Martin and Tzanetakis, 2018). The incidence of six viruses in kiwifruit was studied, including one new species AcCRaV (Zhao et al., 2019).

Five publications focused on co-infection synergisms between different virus or viroid species. The citing number was larger than the number of publications, while simultaneously citing several new species. For example, one publication included five new viruses in their analysis of co-infection patterns in peach (NSPaVF, NeVM, PLPaV, PaLV, and PeVD; Jo et al., 2018).

The citing number was also much larger than the number of review publications. Five systematic literature publications of the European Food Safety Authority performed a listing of non-EU and pest categorization, which were classified into the category of risk assessment, and cited 32 new species, including seven pome, 12 stone, eight ribes, and five rubus viruses. Seven of them (ApNMV, TFDaV, CRMaV, CTLaV, BCaRV, BCLCaV, and RAVA) satisfied the criteria to be considered as Union quarantine pests. With the exception of the impact in the EU territory, on which the Panel was unable to conclude, 11 species (CPrV, MuVA, NSPaV, NeVM, PLPaV, PeVD, PrVF, PrVT, BVBaV, BVE, and BVF) satisfied the other criteria to be considered as potential Union quarantine pests. AGCaV satisfied the criteria to be considered as Union quarantine pests with the possible exception of being absent from the EU territory or having a restricted presence and being under official control. PrGVA met the criterion of having a negative impact in the EU. For those recently discovered, the categorization is associated with high uncertainties, mainly because of the absence of data on their biology, distribution, and impact (Bragard et al., 2019a,b,c,d, 2020).

## Evaluation of a Previous Framework for Biological Characteristics

**FIGURE 3:**
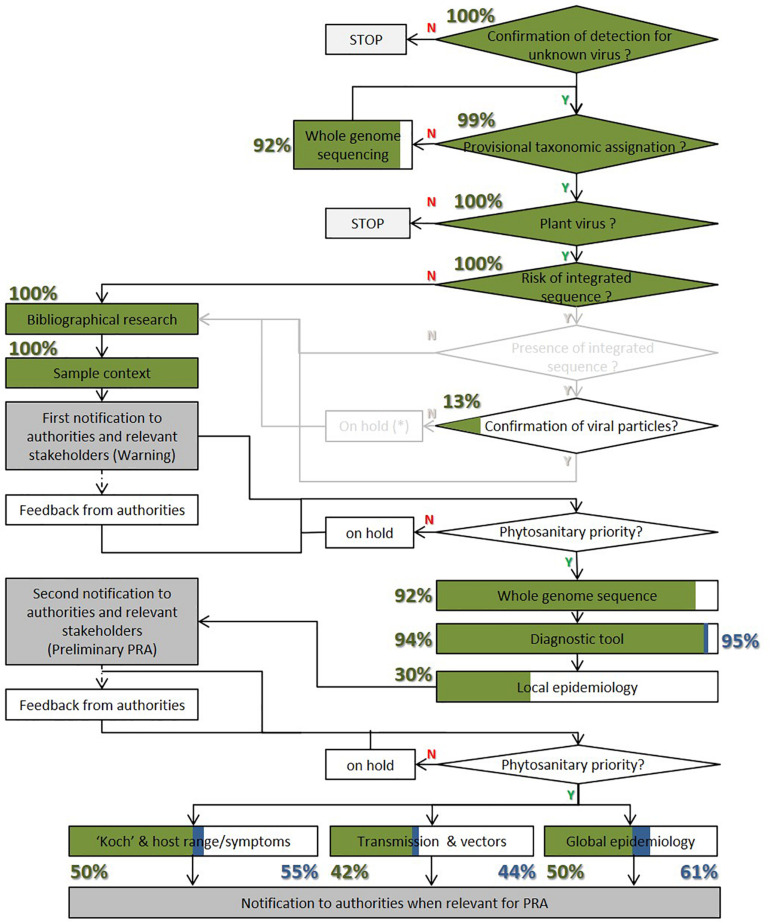
Fulfillment evaluation of a previous published framework for efficient characterization of new phytoviruses (Massart et al., 2017) for the newly discovered fruit tree viruses. Green box and percentage numbers represent the rate of framework completion in the first publication of a new fruit tree virus. Blue box and percentage numbers represent the rate of framework completion for the citing publications and the original one.

The information provided in the first publication of a fruit tree virus and the citing publications was compared to a recently proposed framework for the efficient characterization of new phytoviruses (Massart et al., 2017). This framework proposed a three-step biological characterization of a new plant virus with regular exchanges with the phytosanitary authorities. Figure 3 shows how this framework has been completed for the viruses analyzed in this review in the original publications and those citing them.

### Framework Completion When Publishing a New Fruit Tree Virus

As shown in Figure 3, the rate of framework completion is very high for all the information needed during the first step. Only one publication failed to confirm the detection, while all the other publications described the sample context. A bibliographical search was always carried out but with differences between publications. Some publications explored the biology of related viruses while others did not. The genome integration was never studied because the identified viruses did not present any risk of integrated sequences in the host genome, which is particularly important for the *Caulimoviridae* family. Even in the absence of possible integration, the presence of virions was observed by electron microscopy for 11 species, representing 13% of the publications.

Regarding the second step, nearly all the original publications already sequenced the full genome (92%) and designed a diagnostic test (94%) for the new viral species. Meanwhile, only 30% performed a local epidemiological survey. This low percentage is nevertheless compensated by the fact that half of the publications completed a large-scale survey, among which 37% were only at large scale. This means that an epidemiological survey was carried out in 67% of the first publications. So, the rate of completion of this second step is also very high with the first publication.

For the third step, half of the publications (42) analyzed the symptoms or host range. An analysis of symptoms was carried out for 30 species. This includes the evaluation of symptom association during the epidemiological survey for 21 species (25%); virus incidence in symptomatic and asymptomatic plants) and the observation of symptoms on inoculated plants for 16 species (19%). For eight species, both analyses were carried out. The host range was evaluated for 19 species (23%). The host range was completed by symptom analysis for seven species. The transmission mode was studied for 35 viruses (42%). It was carried out either successfully with indicator plants (12 viruses, 14%), host plant candidates, or other cultivars of the same species (30 viruses, 36%) by mechanical transmission, grafting, or possible vectors (for six species). For eight species, both indicator plants and crop species were inoculated. The seed transmission was only studied for one species (BBLV). As expected, the completion rate of the third step of the framework is, therefore, significantly lower than the two first steps.

### Additional Characterization Performed by the Citing Publications

Figure 3 shows that the improvement in the framework fulfillment by the citing publication was variable depending on the steps, but overall progressed percentage remained low. The information category that was most often covered by the citing publication involved improving the knowledge of the diagnostic method in 14 species. Among them only one species BVE (1%) did not have a published diagnostic protocol or primers in the first publication.

In addition, new progress was made regarding global epidemiology with the report of a new country or continent for nine viruses (11%): AcV-1, PeCV, PeVA, CVEV, CYVCV, CCDaV, PaLV, CRMaV, and NSPaV; which only tested samples collected from the discovery location in the first publications.

A new host was reported for eight species, and only four of them (5%), AGCaV, ApNMV, AVCaV, and NSPaV, did not have their host range tested in the first publications. The other four had different host species identified when first published. New progress of transmission and vectors had been obtained for four species. Two of them (2%), CYVCV and BNRBV, were not included in any transmission experiments when first published. The other two, CiLV-C2 and CiLV-N, had identified possible natural vectors in the first publications. No new species progressed with symptomatology in the citing publications when compared to the first publications.

## Conclusion

This large-scale, retrospective analysis of the biological information provided by the publications reporting the discovery of new fruit tree viruses and their citing publications highlights important trends in the characterization of these new viruses with the publication of the genome sequence.

First, various categories of information are always or nearly always provided: full or nearly full genome, confirmation of detection by an independent technique, and the design of diagnostics primers. Electron microscopy has been carried out in 13% of the publications. Even if this information was scientifically sound and demonstrated the presence of a viral particle, it provided limited information on the biology of the virus and the associated risks for plant health. An epidemiological survey is carried out in more than two-thirds of the publications. However, its extent was highly variable in geographical range, from only the same orchard to several continents and the number of analyzed trees, from a few to hundreds.

Further biological characterization experiments (transmission, host range, and symptom) were only selectively carried out in the publications. For 20% of the discovered species, there was no information at all on the biological characterization, and the publications focused on genetic information. Biological characterization requires more resources and a longer time than genetic characterization. The association of the new species with symptoms might be limited by the low prevalence of symptomatic plants in a survey or the difficulty in reproducing symptoms experimentally. Symptom variability due to cultivar or environmental effects is also a bottleneck to associating a new virus with a disease. In addition, it can be further complicated by the presence of co-infections with other viruses in the orchards.

Therefore, there is a trade-off between quickly publishing partial information and building a robust characterization of a newly identified virus before publishing. Journals have very different policies on this subject. For example, a recent publication presenting only the contigs generated without any biological confirmation has been recently accepted (Wright et al., 2020), while other journals require biological characterization experiments for accepting the publication of a new virus. Whatever the policy of the journal, our retrospective analysis suggests that in more than 90% of the cases, the confirmation of the detection of the new virus by an independent technique and the characterization of the full or nearly full genome have been presented for a peer-review publication of a new fruit tree virus. This information can be recommended as the very minimal information needed for publication. Another important element that has been neglected is the availability of the HTS raw data supporting the virus discovery in public databases to allow the further use of these data by the scientific community.

Beyond the minimal recommendation for scientific publication, an epidemiological survey will provide very useful information with minimal resources and time compared to biological experiments and should be highly recommended. The surveys would indicate the prevalence of the virus in the orchards and several regions/countries. For example, a very recent publication showed that a new foveavirus on *Rubus* spp. was restricted to a single province in Turkey (Gazel et al., 2020). In addition, the epidemiological survey can identify new hosts (Basso et al., 2015) and study the association of the virus with symptoms (Fox, 2020). So, the survey should include healthy trees as well as symptomatic trees, presenting either the same symptoms as the original tree or not. In addition, testing other viruses during the survey is recommended, provided there is a risk of co-infection that could puzzle the analysis of the results.

The lack of resources can be partially solved by improving pre-publication data sharing between research groups. Indeed, a much richer biological characterization could be achieved when a virus is detected by several groups from different host plant presenting diverse symptoms and in several countries using multiple tests and sequencing strategies. This can give quickly valuable information on the host range, field symptomatology and geographical spread of the virus. In addition, such collaborations significantly reduce the burden of publications and minimizes the collective effort to publish new data as the confirmatory experiment and the biological characterization experiments (host range, transmission, and symptomatology) can be shared between partners. For example, HTS data sharing allowed for a better understanding of the geographical spread of a new closterovirus (Koloniuk et al., 2018b). The pre-publication data sharing could also include biological experiments, as was recently shown for the European wheat striate mosaic virus (Sõmera et al., 2020). Overall, pre-publication data sharing enables better risk evaluation and can limit the risks of unnecessary regulatory action.

Our analysis also showed that, in general, the citing publications provided very little additional information on the biological characterization of a newly discovered virus, except for enlarging the geographical spread (11%) or host range (5%). The biological characterization of a newly discovered virus is therefore rarely pursued, which reinforces the need to provide as much information as possible when publishing a new virus, even though the biological characterization experiments can be time-consuming and could delay the publication of the results.

Our analysis also allowed a preliminary evaluation of a published framework for the biological characterization of new plant viruses (Massart et al., 2017) with the reality of publications. According to the current situation with fruit tree viruses, the first step, including discovery, confirmation, taxonomic assignation, and sample documentation, and the second step, corresponding to whole-genome sequencing, diagnostic test development, and local epidemiology, could be gathered together in a single step before publication. The necessity to share both the genome sequence and the raw data, either publicly after publication or within an informal consortium before the publication, should be emphasized. In addition, the host range evaluation should be considered independently from the symptomatology as symptom expression could be very variable and difficult to analyze compared to the ability of the virus to infect a plant. Finally, the analysis of symptoms should include both biological experiments in controlled conditions and association studies from field surveys as proposed in the framework.

## Author Contributions

All the authors significantly contributed to the writing and editing of the manuscript. WH drafted the manuscript and created the figures. SM and SL coordinated the writing and editing of the manuscript.

## Conflict of Interest

The authors declare that the research was conducted in the absence of any commercial or financial relationships that could be construed as a potential conflict of interest.

## Supplementary Material

Supplementary Table 1 Publications reporting a new fruit tree virus.

Supplementary Table 2 Citing publications of a new fruit tree virus.

Supplementary Table 3 Summary of the new viruses infecting fruit trees.
